# Antibacterial Activity of *Lantana camara* Linn and *Lantana montevidensis* Brig Extracts from Cariri-Ceará, Brazil

**DOI:** 10.4103/0975-1483.62211

**Published:** 2010

**Authors:** FS Barreto, EO Sousa, AR Campos, JGM Costa, FFG Rodrigues

**Affiliations:** *Departamento de Química-Biológica, Laboratório de Pesquisa de Produtos Naturais, Universidade Regional do Cariri, Rua Cel. Antônio Luiz 1161, Pimenta, 63105 - 000, Crato-CE, Brazil*; 1 *Vice-Reitoria de Pesquisa e Pós-Graduação, Universidade de Fortaleza, Av. Washington Soares 1321, Edson Queiroz, 60811-905, Fortaleza-CE, Brazil*

**Keywords:** *Lantana camara*, *Lantana montevidensis*, antibacterial activity, microdilution

## Abstract

The use of medicinal plants with therapeutics properties represents a secular tradition in different cultures, mainly in underdeveloped countries. *Lantana camara* Linn and *Lantana montevidensis* Briq (Verbenaceae) found in tropical and subtropical areas around the world are popularly known as “camará” or “chumbinho.” In popular medicines, both plants are used as antipyretic and carminative and in the treatment of respiratory system infections. In this study, the antibacterial activity of the ethanolic extracts of *L. camara* and *L. montevidensis* leaves and roots against gram-positive and gram-negative strains standard and multi-resistant bacteria isolated from clinical material are presented. In order to determine the minimal inhibitory concentration (MIC), the microdilution method was used. The extracts demonstrated antibacterial activity against all tested bacteria, but the *L. montevidensis* fresh leaves extract present the best result against *P. aeruginosa* (MIC 8 μg/mL) and against multi-resistant *E. coli* (Ec 27) (MIC 16 μg/mL). These results drive new researches with both species in order to isolate the constituents responsible for the activity.

## INTRODUCTION

The use of medicinal plants with therapeutical purposes represents a secular tradition in different cultures. Vegetal species have been shown effective to treat many infections, such as tumors[1] Considering the fact that several microorganisms become resistant to conventional antibiotics and the Brazilian vegetal biodiversity, research groups have paid attention to natural products as source of new molecules with pharmacological potential that could be more efficient against nosocomial pathogens and less toxic to human body.[2–4]

The Verbenaceae family comprises 100 genera and about 2600 species distributed in tropical and subtropical regions around the world.[5] *Lantana* is a genus of about 150 species of perennial flowering plants popularly used as antirheumatic, stimulant, antibacterial, biologic control, and as ornamental plant.[6,7]

*Lantana camara* Linn, typical in Americas and Africa, and *Lantana montevidensis* Briq, native to Brazil and Uruguay are popularly known as “camará”, “cambará” or “chumbinho.” They are shrubs introduced in many countries as ornamental plants and considered as invasive species in many parts of the world.[8] In popular medicine, both species are used as carminative, antispasmodic, antiemetic, and to treat respiratory infections as cough, cold, asthma, and bronchitis. Previous studies related antitumoral, antifungal, antimalarial, analgesic, and hepatotoxic activities.[9]

The purpose of this study was to evaluate the *in vitro* antibacterial activity of ethanolic extracts from *L. camara* and *L. montevidensis* leaves and roots using the microdilution method to assay the susceptibilities of five bacteria strains (American Type Culture Collection - ATCC) and two multi-resistant strains isolated from clinical material.

## MATERIALS AND METHODS

### Plant material

The leaves of *L. camara* and *L. montevidensis* were collected in March, 2009, from the Small Aromatic and Medicinal Plants Garden of the Natural Products Research Laboratory (LPPN) at Universidade Regional do Cariri (URCA), city of Crato, Ceará state, Brazil. The exsiccate was deposited at the Herbarium Caririensis “Dárdano de Andrade Lima”, Biology Department, under registry numbers 1662 and 1619, respectively, for *L. camara* and *L. montevidensis*.

### Extracts preparation

Ethanolic extracts were prepared using the cold extraction method. *L. camara* (240 g of the fresh leaves and 185 g of the roots) and *L. montevidensis* (400 g of the fresh leaves and 714 g of the roots) were placed in a flask containing cold ethanol and left in this position for 72 h at ambient temperature. A rotary vacuum pump extractor was used to remove the ethanol from the extracts (under reduced pressure). The extracts were weighted and stored.

### Antibacterial assays

The antibacterial activities of the extracts were investigated by employing a microdilution method, recommended by NCCCLS M7-A6.[10] The assay was carried out with five bacterial species obtained from Fundação Oswaldo Cruz-FIOCRUZ: *Staphylococcus aureus* (ATCC 12692), *Proteus vulgaris* (ATCC 13135), *Pseudomonas aeruginosa* (ATCC 15442), *Víbrio cholareae* (ATCC 15748), *Escherichia coli* (ATCC 2992) and two multiresistant strains obtained from clinical material: *E. coli* (Ec 27, from sputum) and *S. aureus* (Sa 358, from cirurgical wound).

Brain Hear Infusion Broth (BHI 3.8%) was used for bacterial growth (24 h, 35 ± 2°C). The inoculum was an overnight culture of each bacterial species in BHI broth diluted in the same media to a final concentration of approximately 1 × 10^8^ UFC/mL (0.5 nephelometric turbidity units - McFarland scale). After this, the suspension was diluted to 1 × 10^6^ UFC/mL in 10% BHI. 100 μL of each dilution were distributed in 96-well plates plus extracts in different concentrations, achieving 5 × 10^5^ UFC/mL as the final concentration of the inoculum.

Extracts were dissolved in distilled water and dimethyl sulfoxide (DMSO) to a concentration of 103 μg/mL. Further serial dilutions were performed by addition of BHI broth to reach a final concentration in the range of 512 a 8 μg/mL. All experiments were performed in triplicate, and the microdilution trays were incubated at 35 ± 2°C for 24 h. Antibacterial activity was detected using a colorimetric method by adding 25 μL of resauzurin staining (0.01%) aqueous solution in each well at the end of the incubation period. The minimal inhibitory concentration (MIC) was defined as the lowest essential oil concentration able to inhibit the bacteria growth, as indicated by resauzurin staining (bacteria died cells are not able to change the staining color by visual observation-blue to red).

## RESULTS AND DISCUSSION

Several new antibacterial agents are currently being developed in response to the emergence of bacterial resistance to existing drug. New vegetal sources presenting antimicrobial activity and low toxicity could be a viable alternative, with low cost and easily accessible to poor communities where the species are found.[11] The antibacterial activity of ethanolic extract from *L. camara* and *L. montevidensis* presented excellent results against the pathogenic microorganisms tested [Table 1].

**Table 1:** Antibacterial activity of *L. camara* and *L. montevidensis* extracts

Microorganisms	MIC (μg/mL)			
	*L. camara*		*L. montevidensis*	
	LE	RE	LE	RE
*E. coli* (ATCC 25922)	256	512	32	512
*P. vulgaris* (ATCC 13315)	128	64	32	512
*P. aeruginosa* (ATCC 15442)	256	128	8	256
*V. cholareae* (ATCC 15748)	128	≥1024	64	≥1024
*S. aureus* (ATCC 12692)	≥1024	≥1024	128	256
*E. coli* (Ec 27)	256	≥1024	16	≥1024
*S. aureus* (Sa 358)	512	≥1024	128	≥1024

LE = Leaves extract; RE = Roots extract

The extracts presented antibacterial activity against clinically relevant pathogens (gram positive and gram negative). *L. camara* leaves extract was active against *P. vulgaris* and *V. cholerae* (MIC 128 μg/mL for both strains); in addition the root extract was effective against *P. vulgaris* and *P. aeruginosa* (64 and 128 μg/mL, respectively). The leaves and roots *L. montevidensis* extracts were active against *P. vulgaris* and *P. aeruginosa* (MIC 8 μg/mL) and two strains of *E. coli* (MIC 16 μg/mL for the multiresistant strain) as shown in Table 1.

Previous studies using extracts from *Lantana* species showed that they were able to inhibit the growth of gram-positive bacteria strains.[12] However, in this study, the antibacterial activity against gram-negative bacteria was verified, mainly *P. aeruginosa*. This is relevant information as Navon-Venezia (2005)[13] reported that since 1980, after the introduction of carbapenems, no new antibiotics have been used for the treatment of infections caused by multiresistant gram-negative bacilli (*P. aeruginosa*, for example).

The results that we present here are relevant, as the literature has shown that gram-positive bacteria are more sensitive to antibiotics. The gram-negative bacteria display some particularities that inhibit antibiotics penetration, as the lipopolyssacaride layer that determines the permeability and susceptibility to antibiotics.[14] We suggest that data obtained here may suffer seasonal influence and/or be associated with the presence of chemical compounds (terpenes, triterpenes, quinones, alkaloids, and flavonoids) derived from *Lantana* species secondary metabolism as reported in phytochemical studies.
